# High-temperature operation of broadband bidirectional terahertz quantum-cascade lasers

**DOI:** 10.1038/srep32978

**Published:** 2016-09-12

**Authors:** Sudeep Khanal, Liang Gao, Le Zhao, John L. Reno, Sushil Kumar

**Affiliations:** 1 Department of Electrical and Computer Engineering, Lehigh University, Bethlehem, PA 18015, USA; 2 Sandia National Laboratories, Center of Integrated Nanotechnologies, MS 1303, Albuquerque, NM 87185, USA

## Abstract

Terahertz quantum cascade lasers (QCLs) with a broadband gain medium could play an important role for sensing and spectroscopy since then distributed-feedback schemes could be utilized to produce laser arrays on a single semiconductor chip with wide spectral coverage. QCLs can be designed to emit at two different frequencies when biased with opposing electrical polarities. Here, terahertz QCLs with bidirectional operation are developed to achieve broadband lasing from the same semiconductor chip. A three-well design scheme with shallow-well GaAs/Al_0.10_Ga_0.90_As superlattices is developed to achieve high-temperature operation for bidirectional QCLs. It is shown that shallow-well heterostructures lead to optimal quantum-transport in the superlattice for bidirectional operation compared to the prevalent GaAs/Al_0.15_Ga_0.85_As material system. Broadband lasing in the frequency range of 3.1–3.7 THz is demonstrated for one QCL design, which achieves maximum operating temperatures of 147 K and 128 K respectively in opposing polarities. Dual-color lasing with large frequency separation is demonstrated for a second QCL, that emits at ~3.7 THz and operates up to 121 K in one polarity, and at ~2.7 THz up to 105 K in the opposing polarity. These are the highest operating temperatures achieved for broadband terahertz QCLs at the respective emission frequencies, and could lead to commercial development of broadband terahertz laser arrays.

Numerous potential applications exist for spectroscopy and sensing in the terahertz range of the electromagnetic spectrum (*ν* ~ 1–10 THz, *λ* ~ 30–300 *μ*m), since large polar molecules have characteristic spectral signatures across this frequency range. To perform terahertz spectroscopy of such molecules it is desirable to have high-power sources of coherent radiation that could be tuned across a large bandwidth. Terahertz quantum cascade lasers (QCLs)^1^ are the most powerful solid-state sources of terahertz radiation that are uniquely poised to target a range of potential applications in fields as diverse as astronomy, atmospheric science, biology, medicine, and chemical analysis. Terahertz QCLs could be designed to emit output power in the range tens of milliwatt at cryogenic temperatures. The output power at the desired operating temperature and other properties such as the spectral and radiative characteristics depend on the design scheme of the active region as well as that of the cavity. QCLs with best operating temperatures are now developed using three-well design schemes in the GaAs/Al_0.15_Ga_0.85_As material system^2^,^3^ with an underlying characteristic featuring resonant-phonon depopulation^4^ and one-well injection^5^ schemes.

Terahertz QCLs are typically desired for broadband spectroscopy for which it would be useful to have semiconductor chips with single-mode QCL arrays that could provide wide spectral coverage. Such laser arrays have been developed for mid-infrared (IR) QCLs^6^,^7^ and are now commercially available for mid-IR frequencies. In general, a typical terahertz QCL provides gain bandwidth of ~0.2–0.4 THz around its central emission frequency. It is desirable to increase the bandwidth to ~1 THz to access broad spectral features for spectral fingerprinting. In that context, it is helpful to consider prior work on development of multi-color or broadband terahertz QCLs, which is summarized in chronological order in Table 1. Heterogeneous cascade design schemes have been used to develop broadband terahertz QCLs^8^,^9^,^10^; however, the increase in bandwidth comes at the cost of reduced gain since effectively smaller number of QCL modules produce gain at a given frequency. Hence, thicker active regions may be required, which is challenging for epitaxial growth. Additionally, such designs are challenging and may require multiple growth iterations, since inter-stack current-transport needs to be properly matched for a given bias field to avoid instabilities due to electric-field domain formation. Dual-color lasing was also realized in ref. 11 due to two separate intersubband radiative transitions within the same quantum-wells of the superlattice. However, it was argued that such a lasing characteristic is detrimental to the QCL operation, and it is better to suppress multi-photon transitions from the same wells to maintain stable electrical operation in the QCL^12^. A terahertz QCL with large bandwidth was also realized by using strong tunnel-coupling for injection transport in a two-well design^13^. However, such a strategy is less likely to work for resonant-phonon design schemes, which suffer from large parasitic currents if thin injector barriers (large tunnel-coupling) are used.

One of the unique features of QCLs is that they could be designed for lasing independently at two different frequencies for positive and negative electrical bias respectively^14^. This is possible because of the unipolar carrier transport in intersubband lasers. The gain bandwidth for lasing in resonant-phonon terahertz QCLs is typically in the range of 0.2–0.5 THz. When properly designed, bidirectional QCLs could potentially increase the gain bandwidth to ~1 THz without compromising the performance characteristics significantly, since entire gain is available for a given operating polarity. There have been very few reports of bidirectional terahertz QCLs. The first bidirectional terahertz QCLs were demonstrated with a four-well GaAs/Al_0.15_Ga_0.85_As design scheme with one-well injector and resonant-phonon depopulation^15^,^16^. In this work, we show that improved performance could be realized for bidirectional QCLs when they are implemented with the more robust three-well design scheme; however, in the following, it is argued that shallow-wells (with 10%–Al barriers) need be employed to achieve the design flexibility that is needed for achieving gain at separate frequencies in opposing polarities. For such designs, the maximum operating temperature is better or at-par with that of any other previously developed broadband terahertz QCLs. Shallow-wells also lead to lower threshold current-densities compared to the threshold current-densities in three-well terahertz QCLs that are predominantly based on 15%–Al barriers^17^, which is an additional benefit in the presented designs.

## Results

### Design parameters for three-well bandstructure

To understand the importance of utilizing shallow-wells in a three-well bidirectional QCL design as proposed here, it is instructive to know the typical design parameters for three-well terahertz QCLs with best temperature performances, which are listed in Table 2. QCLs with variable-height barriers^18^,^19^ (that are still primarily based on GaAs/Al_0.15_Ga_0.85_As heterostructures) are not included in the table since their design parameters are similar to that in ref. 3. Arguably, the two most important design parameters for such resonant-phonon designs are the tunneling parameters Δ_inj_ and Δ_col_, which refer to the energy-splitting (anticrossing) between the tunneling subbands when they are resonantly aligned for injection and extraction respectively. As is evident from the compilation in the table, optimum transport is achieved for Δ_inj_ ~ 2–2.5 meV and Δ_col_ ~ 4–5 meV. The degree of diagonality of the radiative transition is represented by the oscillator-strength *f*_osc_; however, *f*_osc_ primarily impacts the operating current-densities^20^ while not affecting the maximum operating temperature *T*_max_^3^ sensitively. In general, the coupling strength needs to be reduced by utilizing thicker tunnel barriers for more “vertical” (large *f*_osc_) designs lest the low-bias parasitic current-density becomes too large to allow the superlattice to reach the required bias alignment for lasing.

### Shallow-wells for bidirectional resonant-tunneling

**Figure 1:**
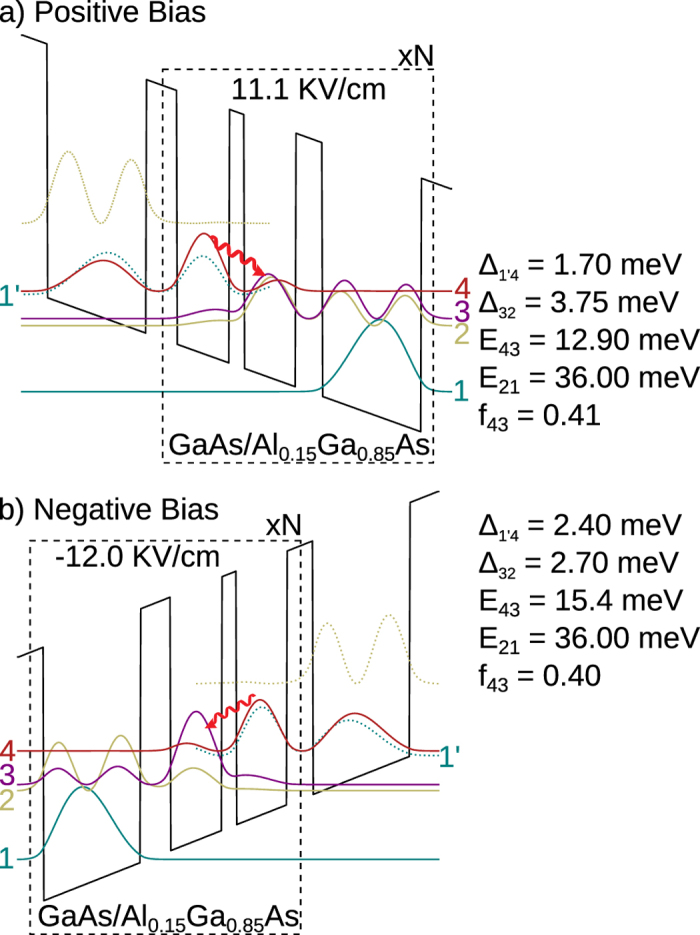
One-period conduction band-diagrams for a hypothetical three-well bidirectional QCL design with 15%-Al barriers. Plots in both polarities are shown for the “design-bias” corresponding to the injection resonance (1′–4) alignment. Starting from the thickest barrier in the repeat period, the layer thicknesses in nm are (barriers are indicated in bold-font) **5**.**2**/8.9/**2**.**5**/8.8/**4**.**5**/16.8. (**a**) **Positive Bias**: 52 mV/period, Δ_inj_ ≡ Δ_1′4_ = 1.7 meV, Δ_col_ ≡ Δ_32_ = 3.75 meV. (**b**) **Negative Bias**: 56 mV/period, Δ_inj_ ≡ Δ_1′4_ = 2.4 meV, Δ_col_ ≡ Δ_32_ = 2.7 meV.

Figure 1 shows a hypothetical design for a three-well bidirectional terahertz QCL structure in GaAs/Al_0.15_Ga_0.85_As. It is designed for optical gain centered around frequencies of 3.1 THz (*E*_43_ ~ 12.9 meV) and 3.7 THz (*E*_43_ ~ 15.4 meV) in positive and negative polarity operation respectively. The wide-well serves as the injector-well in both polarities; however, the role of the two narrower wells is interchanged in opposing polarities, which bring the required asymmetry to the design. Similarly, the roles of injection and extraction tunnel barriers is interchanged with the operating polarity. The purpose of the design in Fig. 1 is to show that when tunneling parameters Δ_inj_ and Δ_col_ are optimized for one polarity (in this case, for positive-bias as in Fig. 1a), the corresponding values in opposing polarity veer away from that optimally desired as per Table 2. This is because, in general, for designs with relatively deep wells including that with GaAs/Al_0.15_Ga_0.85_As heterostructures, the injector barrier (the 5.2 nm thick barrier) needs to be kept thicker in comparison to the extraction barrier (the 4.5 nm thick barrier) to keep 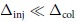, which could only be realized for one polarity. While we did not grow and test the structure presented in Fig. 1, it will most likely perform well only in positive-polarity. In reverse-polarity the performance is likely to degrade significantly in comparison owing to a large parasitic current (due to large Δ_inj_ ~ 2.4 meV) and poor extraction efficiency (due to small Δ_col_ ~ 2.7 meV).

#### Broadband bidirectional QCL (Design A)

**Figure 2:**
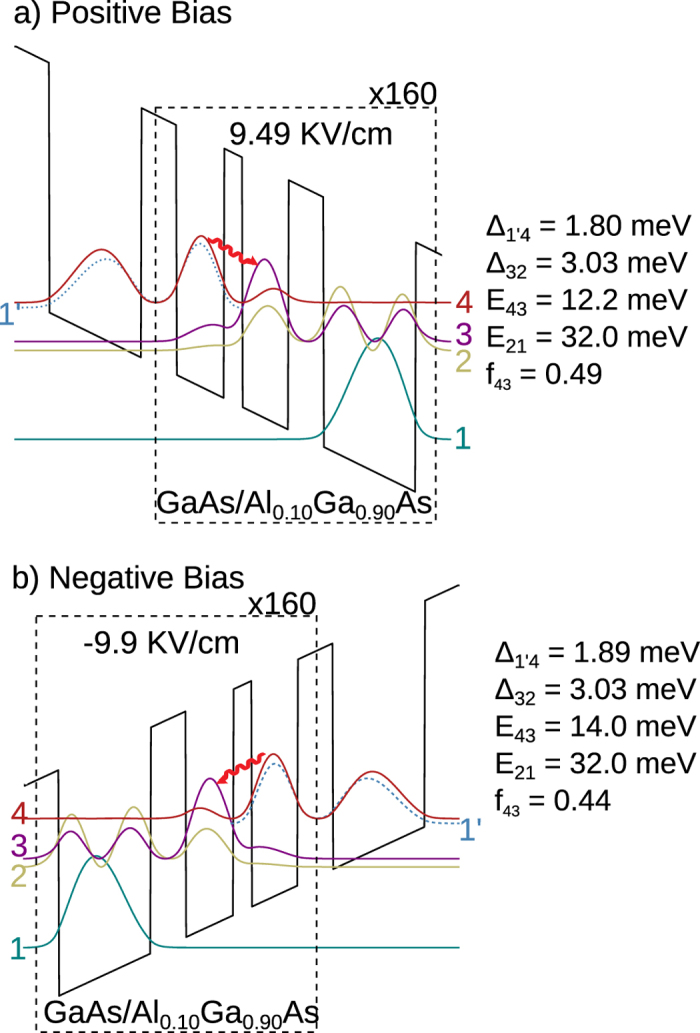
Band-diagrams for the re-designed three-well bidirectional QCL with 10%-Al barriers, which was experimentally realized. The design is named BIDR3W160, or “**Design A**” for purposes of discussion. Plots for injection-resonance are shown. The center frequencies of radiative transitions differ by ~0.4 THz in opposite polarities (difference in radiative energy ~1.8 meV). Starting from the thickest barrier in the repeat period, the layer thicknesses in nm are (barriers are indicated in bold-font) **6**.**5**/8.76/**3**.**39**/8.48/**6**.**5**/16.96. Each period is delta-doped at 2.7 × 10^10^ cm^−2^ at the center of the widest well, which corresponds to an average doping 5.3 × 10^15^ cm^−3^ in the QCL. (**a**) **Positive Bias**: 48 mV/period, Δ_inj_ ≡ Δ_1′4_ = 1.8 meV, Δ_col_ ≡ Δ_32_ = 3.0 meV. (**b**) **Negative Bias**: 50 mV/period,Δ_inj_ ≡ Δ_1′4_ = 1.9 meV, Δ_col_ ≡ Δ_32_ = 3.0 meV.

The undesired asymmetry for tunneling parameters in opposite polarities for designs based on GaAs/Al_0.15_Ga_0.85_As could be avoided by utilization of shallow-well heterostructures. With deep-wells, the extraction barrier has to be thinner than the injection barrier for aforementioned reasons. However, for shallow-wells, the extraction barrier needs to be made thicker than usual, because now the extraction subbands are energetically located closer to the continuum in the injector well, which requires use of a thicker-than-usual tunnel barrier to maintain the desired tunnel coupling Δ_col_. Consequently, for a shallow-well QCL design, the thickness of the injection and extraction barriers could be kept similar while still maintaining desired Δ_inj_ and Δ_col_. Figure 2 shows the re-designed three-well bidirectional terahertz QCL structure with a GaAs/Al_0.10_Ga_0.90_As superlattice. The barrier height is now lowered to ~90 meV from ~135 meV for GaAs/Al_0.15_Ga_0.85_As. As can be seen from the layer thicknesses, the injection and extraction barriers are now of the same thickness (6.5 nm), and consequently, the tunneling parameters Δ_inj_ ≡ Δ_1′4_ and Δ_col_ ≡ Δ_32_ could be kept similar for both polarities. The design in Fig. 2 (labeled BIDR3W160, or “Design A”) was realized with smaller tunnel couplings compared to that in Table 2, to keep the parasitic leakage current small. The parasitic leakage in general increases for such shallow-well designs due to carrier-scattering into the energy continuum over the barriers^17^. Design A was the first trial for a bidirectional three-well terahertz QCL, hence, the radiative frequencies were made only slightly different in opposite polarities, centered around 3.0 THz and 3.4 THz for positive and negative polarity operation respectively, which allows a design with almost similar design parameters for either polarity including *f*_osc_.

**Figure 3:**
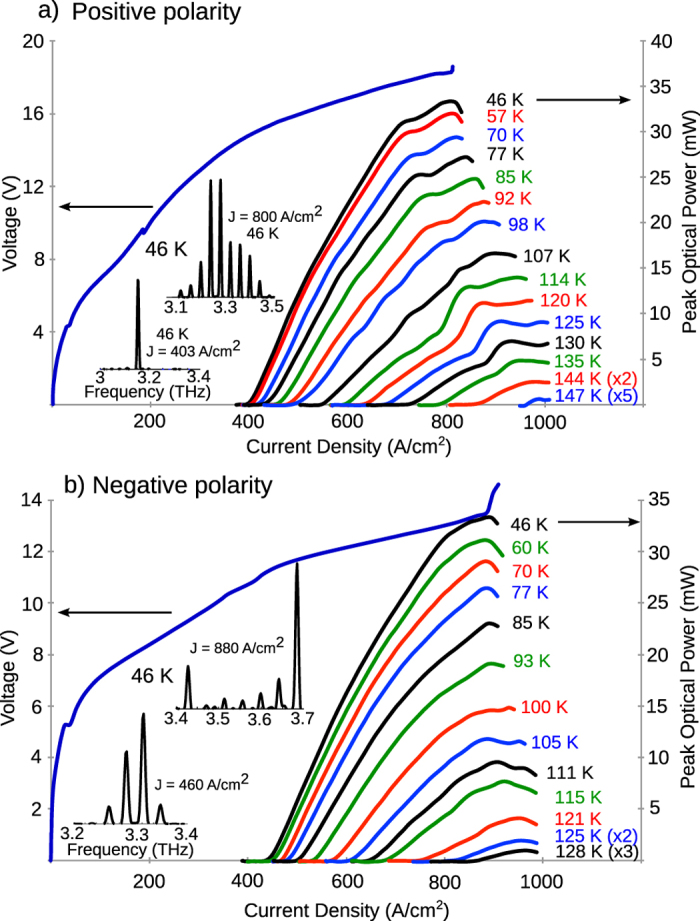
Experimental results from a representative edge-emitting ridge-cavity bidirectional QCL corresponding to Design A. *L*-*I* and *I*-*V* characteristics from a 1.1 mm long and 150 *μ*m wide laser biased in pulsed operation are shown. The measurements were performed with 200 ns pulses repeated at 100 kHz, where data in (**a**) is for QCL biased in positive-polarity on top contact, and (**b**) is for measurement with negative-polarity biasing. The corresponding insets The insets show laser spectra measured in linear-scan mode using a Fourier-transform infrared spectrometer and a room-temperature pyroelectric detector. The QCL’s radiation frequency was centered around ~3.2 THz in positive-polarity bias, and around ~3.5 THz in negative-polarity bias.

Figure 3 shows light-current (*L*-*I*) and current-voltage (*I*-*V*) curves for a representative ridge-cavity QCL for Design A in both polarities. The *L*-*I* were recorded with a room-temperature pyroelectric detector (model number: Gentec THz 2I-BL-BNC with THz-WC-13), and the absolute power was calibrated using a thermopile power meter (model number: Scientech AC2500 with AC25H) as is reported without any corrections to the detected signal. A winston-cone was used in front of the laser’s facet for power collection. This QCL operated up to *T*_max_ ~ 147 K with *J*_th_ ~ 400 A/cm^2^ low-temperature threshold current-density in positive polarity, and up to *T*_max_ ~ 128 K with *J*_th_ ~ 420 A/cm^2^ in negative polarity. Representative spectra for both polarities are also shown as insets; the typical frequency coverage was from 3.1–3.5 THz in positive polarity, and from 3.3–3.7 THz in negative-polarity. The highlights of the experimental results are the wide combined spectral coverage from 3.1–3.7 THz from a single QCL, as was originally intended from such a design. Secondly, the use of shallow-wells leads to low threshold current-densities, compared to typical values for such three-well terahertz QCLs (as in Table 2). The reduced operating current-density is due to weaker interface-roughness scattering in shallow-well superlattices that utilize thicker tunnel barriers^17^, and is a useful practical feature to keep low electric power dissipation for such QCLs in operation. These characteristics are realized without a significant degradation in maximum operating temperatures, which are better than that from all other reports of previously reported broadband terahertz QCLs (as in Table 1).

#### Dual-color bidirectional QCL (Design B)

**Figure 4:**
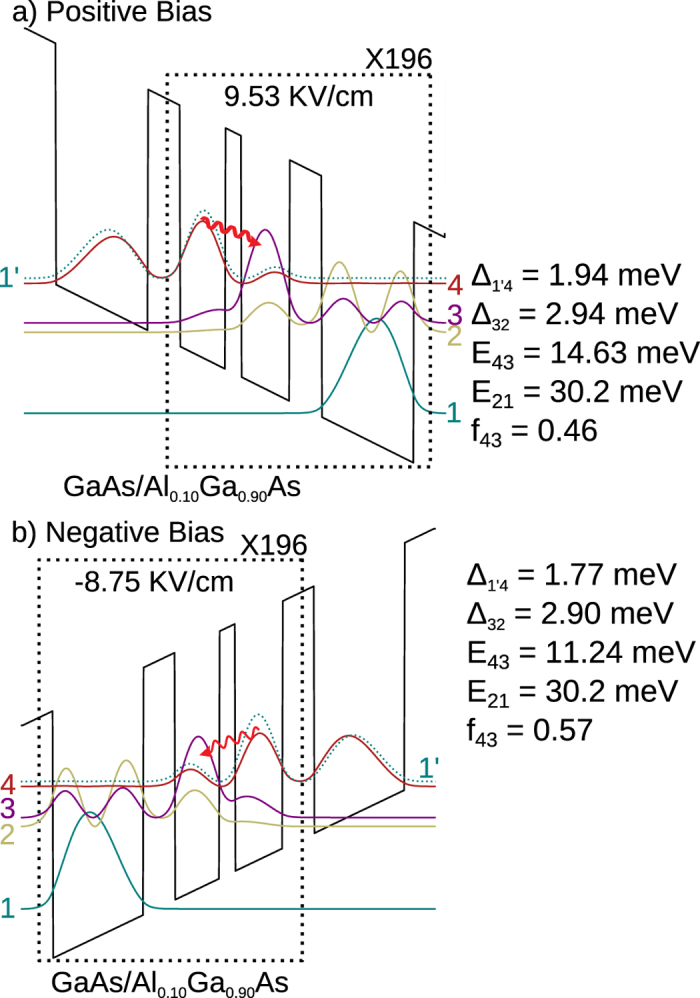
Band-diagrams for the second bidirectional QCL with 10%-Al barriers that was grown and experimentally characterized. The design is named BIDR196B or “**Design B**”. Plots in both polarities are shown for injection resonance, where center frequencies for the radiative transitions are much more widely separated for opposing polarities, by ~0.8 THz (~3.4 meV), compared to Design A from Fig. 2. Starting from the thickest barrier in the repeat period, the layer thicknesses in nm are (barriers are indicated in bold-font) **6**.**22**/8.76/**3**.**11**/9.33/**6**.**22**/17.80. The wide injector well is uniformly doped at 1.7 × 10^16^ cm^−3^, which corresponds to an average doping of 5.9 × 10^15^ cm^−3^ across the superlattice. (**a**) **Positive Bias**: 49 mV/period, Δ_inj_ ≡ Δ_1′4_ = 1.9 meV, Δ_col_ ≡ Δ_32_ = 2.9 meV. (**b**) **Negative Bias**: 45 mV/period, Δ_inj_ ≡ Δ_1′4_ = 1.8 meV, Δ_col_ ≡ Δ_32_ = 2.9 meV.

To show the flexibility of the three-well resonant-phonon design scheme for bidirectional operation at any desired frequencies, a second design was implemented in which the radiative frequency for opposite polarities was significantly separated by ~1 THz. The design (labeled BIDR196B, or “Design B”) is shown in Fig. 4. As with Design A, the injector and the extraction barriers are of the same thickness (6.22 nm) due to the choice of shallow-well heterostructures, which leads to similar tunneling parametersΔ_inj_ and Δ_col_ for either polarity. The increased asymmetry in the radiative frequency is achieved by increasing the asymmetry in the thickness of of the two active-region wells. If the tunneling parameters Δ_inj_ and Δ_col_ have to be kept same, the asymmetry is then reflected primarily in the radiative oscillator strength *f*_osc_, which now becomes different for opposing polarities. However, as mentioned previously, *f*_osc_ affects the temperature performance less sensitively and hence, different *f*_osc_ values do not limit the available design flexibility for bidirectional operation at desired frequencies.

**Figure 5:**
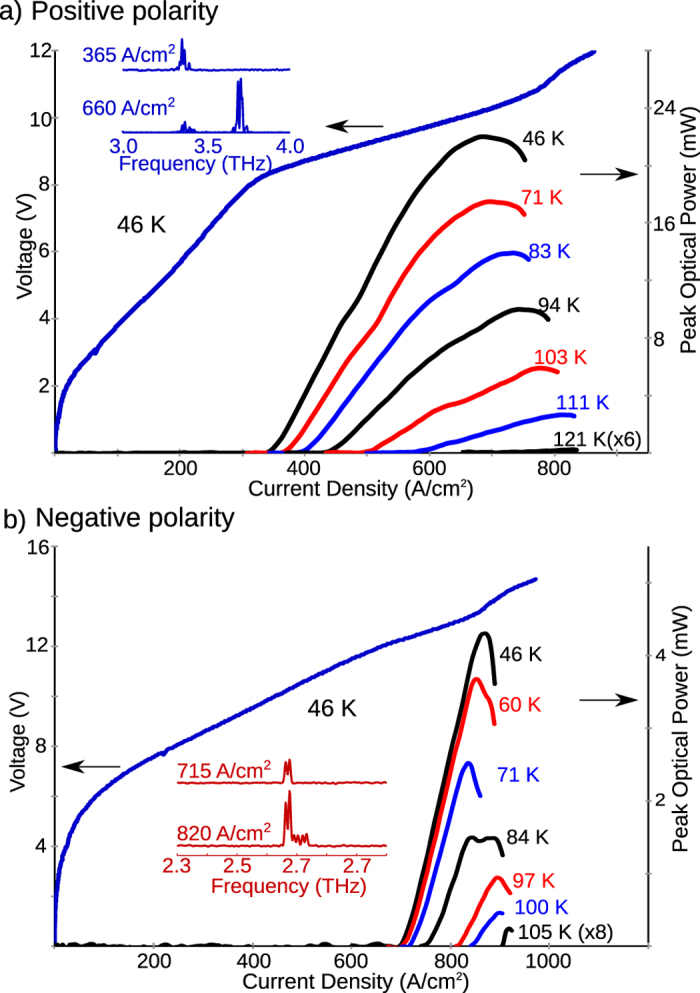
Experimental results from a representative ridge-cavity bidirectional QCL corresponding to Design B. *L*-*I* and *I*-*V* characteristics from a 1.7 mm long and 150 *μ*m wide laser biased in pulsed operation are shown. The QCL’s radiation frequency was centered around 3.7 THz in positive-polarity bias, and around 2.7 THz in negative-polarity bias.

The experimental results from a representative QCL (wafer VB0788) for Design B are shown in Fig. 5. The QCL emitted from 3.4–3.8 THz and operated up to a maximum temperature of 121 K with low-temperature *J*_th_ ~ 360 A/cm^2^ in positive-polarity operation, and emitted from 2.6–2.8 THz and operated up to 105 K with low-temperature *J*_th_ ~ 700 A/cm^2^ in negative-polarity. The higher operating current-density and lower *T*_max_ for negative-polarity operation is due to large parasitic leakage current in the superlattice that is a characteristic of lower radiative-frequency in this polarity. In general, high parasitic current-densities are a characteristic of three-well resonant-phonon QCLs, especially when designed for operation below 3 THz.

## Discussion

In conclusion, we have demonstrated high-temperature operation of broadband/dual-color terahertz QCLs based on the bidirectional three-well resonant-phonon superlattices. Designs based on three-well periods are robust with respect to growth fluctuations and are easier to optimize due to fewer variable parameters in comparison to bandstructures with greater number of quantum-wells. Design flexibility is demonstrated by developing a QCL with wide spectral coverage across a bandwidth of 0.7 THz centered around 3.5 THz for one QCL, whereas dual-color operation is demonstrated for a second bidirectional QCL with ~1 THz frequency separation when the QCL is operated in opposing polarities. The maximum operating temperature is better than that of previously reported broadband and multi-color terahertz QCLs, which were all developed with more number of quantum-wells in the repeated QCL bandstructure. It is argued that the key enabling characteristic of achieving robust bidirectional operation in the three-well structures is the use of shallow-well heterostructures based on GaAs/Al_0.10_Ga_0.90_As, which allows optimization of resonant-tunneling electron-transport in either polarity. Bidirectional terahertz QCLs may be a better alternative to achieve broadband gain at higher temperatures from the same active medium when compared to QCL designs with heterogeneous cascade structures for development of broadband terahertz laser arrays on monolithic semiconductor chips, and for applications in multi-color terahertz spectroscopy and sensing.

## Methods

The bidirectional QCL structure in Design A (BIDR3W160) was grown by molecular beam epitaxy (wafer VB0682) with 160 cascaded periods, leading to an overall thickness of 8 *μ*m. The bidirectional QCL structure in Design B (BIDR196B) was grown by molecular beam epitaxy (wafer VB0788) with 196 cascaded periods, leading to an overall thickness of 10 *μ*m. For operation in both polarities, 10 nm thick GaAs contact layers were grown with 5 × 10^19^ cm^−3^ doping followed by 50 nm thick layers with 5 × 10^18^ cm^−3^ doping on either side of the active superlattice for both wafers. A 300 nm thick Al_0.55_Ga_0.45_As etch-stop layer was grown as a layer preceding the entire stack. Cu-Cu based metallic waveguides were fabricated using standard terahertz QCL fabrication techniques. A sequence of Ta/Cu/Au were deposited as both top (20/200/100 nm) and bottom (20/200/100 nm) metallic layers. Fabry- Pérot ridge cavities were processed by wet-etching using H_2_SO_4_:H_2_O_2_:H_2_O etchant in 1:8:80 concentration. To operate in both polarities, the ridges are processed with highly-doped GaAs contact layers left intact beneath the metal cladding layers of the cavities. Fabricated devices were cleaved and indium soldered on a copper mount, wire-bonded and mounted on the cold-stage of a Stirling cryocooler for characterization.

## Additional Information

**How to cite this article**: Khanal, S. *et al*. High-temperature operation of broadband bidirectional terahertz quantum-cascade lasers. *Sci. Rep.* **6**, 32978; doi: 10.1038/srep32978 (2016).

## Figures and Tables

**Table 1:** Multi-color/broadband terahertz QCLs listed in chronological order of development (for QCLs operating without the assistance of an applied magnetic-field).

Reference	Design Type	*ν*	*T*_max_	*J*_th_
Ref. 8	BTC	2.6 THz3.0 THz	60 K (*ν* = 2.6 THz) 91 K (*ν* = 3 THz)	79 A/cm^2^ (4 K)209 A/cm^2^ (4 K)
Ref. 15	Bidirectional 4-well RP	2.3 THz (forward) 4 THz (reverse)	98 K (forward) 120 K (reverse)	490 A/cm^2^ (forward) (4 K)330 A/cm^2^ (reverse) (4 K)
Ref. 13	2-well	2.8–4 THz	125 K	260 A/cm^2^ (10 K)
Ref. 9	BTC	2.2–3.2 THz	125 K	950 A/cm^2^ (10 K)
Ref. 11	SA	1.8 THz 4 THz	163 K (*ν* = 1.8 THz) 151 K (*ν* = 4 THz)	865 A/cm^2^ (10 K) 325 A/cm^2^ (10 K)
Ref. 10	BTC	2.3–3 THz	118 K	190 A/cm^2^ (10 K)
This work: Design A	Bidirectional 3-well RP	3.2 THz (forward) 3.5 THz (reverse)	147 K (forward) 128 K (reverse)	380 A/cm^2^ (forward) (46 K) 420 A/cm^2^ (reverse) (46 K)
This work: Design B	Bidirectional 3-well RP	3.7 THz (forward) 2.7 THz (reverse)	121 K (forward) 105 K (reverse)	360 A/cm^2^ (forward) (46 K) 700 A/cm^2^ (reverse) (46 K)

Design types such as BTC, RP and SA are acronyms for active-region designs based on “bound-to-continuum”, “resonant-phonon” and “scattering-assisted” schemes respectively. Key operation parameters are listed, where *T*_max_ is the maximum operating temperature in pulsed operation, *J*_th_ is the threshold-current density at the specified temperature, and *ν* is the center-frequency of the lasing spectra from a typical ridge-cavity QCL.

**Table 2:** Key design and operation parameters of previously reported terahertz QCLs based on three-well resonant-phonon design scheme (all listed QCLs are in the GaAs/Al_0.15_Ga_0.85_As material-system).

Reference	*ν*	Δ_inj_ (meV)	Δ_col_ (meV)	*f*_osc_	*T*_max_	*J*_th_
Ref. 21	3.0 THz	1.8 meV	3.8 meV	0.86	178 K	700 A/cm^2^ (78 K)
Ref. 20	3.8 THz	2.2 meV	4.8 meV	0.38	186 K	410 A/cm^2^ (5 K)
Ref. 22	3.1 THz	1.8 meV	3.9 meV	0.85	170 K	700 A/cm^2^ (10 K)
Ref. 3	3.1 THz	1.7 meV	3.9 meV	0.57	200 K	970 A/cm^2^ (8 K)
Ref. 23	4.0 THz	2.5 meV	4.1 meV	0.37	151 K	680 A/cm^2^ (5 K)

QCLs emitting in the range of ~3–4 THz with *T*_max_ > 150 K are listed in chronological order of publication. Δ_inj_ is the energy splitting (anticrossing) at injection resonance, Δ_col_ is the corresponding value for extraction resonance, and *f*_osc_ represents the normalized radiative oscillator-strength at design bias.
